# Seroprevalence of SARS-CoV-2 before/after case zero: Valerio Bordino

**DOI:** 10.1093/eurpub/ckac131.207

**Published:** 2022-10-25

**Authors:** V Bordino, C Vicentini, AR Cornio, D Meddis, CM Zotti

**Affiliations:** Department of Sciences of Public Health and Pediatrics, University of Turin, Turin, Italy

## Abstract

**Introduction:**

Italy was one of the first EU countries hit by the COVID-19 pandemic. Currently, Italy has reported 15.5 million cases of COVID-19 and 161000 deaths. Meanwhile, the vaccination campaign against COVID-19 began in Italy at the end of 2020, using mRNA and viral vector vaccines (immunizing people against Spike protein of SARS-CoV-2. The purpose of this study was to estimate, in a representative sample of the Italian population, the prevalence of antibodies against SARS-CoV2 in 2019 (before case zero, identified in Italy in February 2020) and in 2021, after 3 pandemic waves and a vaccination campaign.

**Methods:**

During October / November 2019: 365 participants were selected in the Piedmontese population among those who went to a hospital for routine blood tests. The population was selected on the basis of age and gender to be representative of the Italian population. The same number of patients was selected in the first quarter of 2021, the inclusion and exclusion criteria remained the same. Sera were searched for spike protein of SARS-CoV-2 and, if positive, tested for anti-nucleocapsid antibodies.

**Results:**

Our preliminary data show that half of the sample for both years is female. In the 2019 sample, i.e. before case zero was identified in Italy (Lombardy), five of the sera (4 males and one female) tested positive for anti-Spike,indicating a previous infection (vaccine didn't exist). In the 2021 sample, 152 males and 139 females tested positive for IgG anti-spike, for a total of 291. The prevalence therefore passed from 1.37% to 79.73%. As regards the search for ANti-Nantibodies, one male and one female tested positive in 2019; in 2021 9 males and 13 females.

**Conclusions:**

The results of our study show that in 2019, before the first official case in Italy was highlighted, coronavirus was already circulating. The prevalence has risen exponentially, going from less than 2% to around 80%.

**Key messages:**

• Covid-19 was circulating in Italy in 2019.

• Seroprevalence of anti-S in 2021 was about 20%.

